# Combination Formulation of Tetrodotoxin and Lidocaine as a Potential Therapy for Severe Arrhythmias

**DOI:** 10.3390/md17120685

**Published:** 2019-12-05

**Authors:** Bihong Hong, Jianlin He, Qingqing Le, Kaikai Bai, Yongqiang Chen, Wenwen Huang

**Affiliations:** 1 Third Institute of Oceanography, Ministry of Natural Resources, Xiamen 361005, China; jlhe@tio.org.cn (J.H.); leqingqing@tio.org.cn (Q.L.); kkbai@tio.org.cn (K.B.); yqchen2016@jmu.edu.cn (Y.C.); wwhuang@tio.org.cn (W.H.); 2 Technical Innovation Center for Utilization of Marine Biological Resources, Ministry of Natural Resources, Xiamen 361005, China; 3 College of Food and Biological Engineering, Jimei University, Xiamen 361021, China

**Keywords:** Tetrodotoxin, lidocaine, combination preparation, arrhythmia, freeze dried powder

## Abstract

Severe arrhythmias—such as ventricular arrhythmias—can be fatal, but treatment options are limited. The effects of a combined formulation of tetrodotoxin (TTX) and lidocaine (LID) on severe arrhythmias were studied. Patch clamp recording data showed that the combination of LID and TTX had a stronger inhibitory effect on voltage-gated sodium channel 1.5 (Nav1.5) than that of either TTX or LID alone. LID + TTX formulations were prepared with optimal stability containing 1 μg of TTX, 5 mg of LID, 6 mg of mannitol, and 4 mg of dextran-40 and then freeze dried. This formulation significantly delayed the onset and shortened the duration of arrhythmia induced by aconitine in rats. Arrhythmia-originated death was avoided by the combined formulation, with a decrease in the mortality rate from 64% to 0%. The data also suggests that the anti-arrhythmic effect of the combination was greater than that of either TTX or LID alone. This paper offers new approaches to develop effective medications against arrhythmias.

## 1. Introduction

Arrhythmias may arise alone or lead to other cardiovascular disorders, sometimes even resulting in heart failure or sudden death. Severe ventricular tachycardia or fibrillation can be fatal, and this occurs when the heart cannot pump blood at a normal pace to provide effective cardiac output. Emergency treatment of ventricular arrhythmias is a challenge, since current medications can lead to side effects such as thyroid dysfunction, pulmonary fibrosis, and anaphylaxis [1,2,3]. Therefore, better medication is needed.

**Figure 1:**
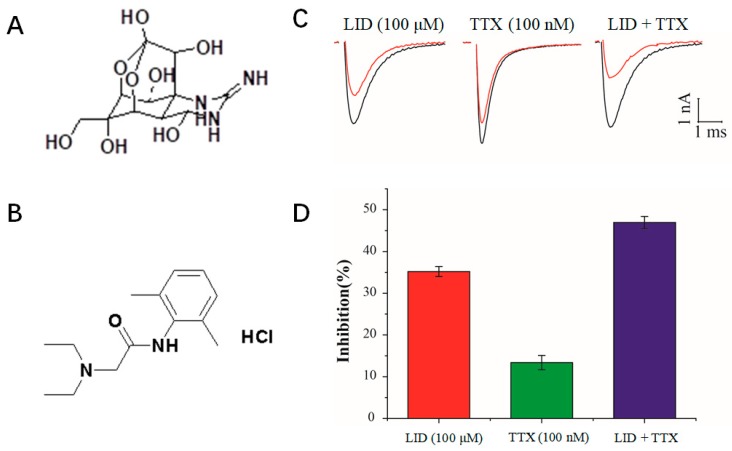
Inhibition of Nav1.5 currents by lidocaine (LID), tetrodotoxin (TTX) and an LID + TTX mixture. The structures of (**A**) TTX and (**B**) LID. (**C**) Representative traces were evoked by a single 15 ms depolarizing pulse to −10 mV from the holding potential of −130 mV in the absence (blank lines) or presence (red lines) of LID (100 μM), TTX (100 nM) or an LID (100 μM) + TTX (100 nM) mixture. (**D**) Inhibition rate of Nav1.5 by LID (100 μM), TTX (100 nM) or an LID (100 μM) + TTX (100 nM) mixture.

Lidocaine (LID, shown in Figure 1A) is a common anti-arrhythmic agent. It is often efficacious when used as a single agent. Increasing the dose of LID might help improve its curative effect. However, LID at a high dose can cause some side effects such as central nervous system disturbances, gastrointestinal distress, and cardiac functional insufficiency. It has been reported that LID used with another drug may have better efficacy and fewer side effects [4,5]. Therefore, much effort has been devoted to studying potential combinations of LID and other medications.

Tetrodotoxin (TTX, shown in Figure 1B), a natural neurotoxin [6] isolated from Tetraodontidae, is considered to have anti-heroin withdrawal, analgesic, anesthetic, respiratory sedation, and anti-arrhythmic properties [7,8,9,10,11,12]. Voltage-gated sodium channels that drive membrane excitability to affect cardiac cell action potentials [9] can be specifically blocked by TTX. Therefore, TTX may exert anti-arrhythmic properties by blocking selective sodium channels without having much influence on potassium or calcium currents. In fact, it was reported that TTX possessed anti-arrhythmic activity in rats subjected to myocardial ischemia by blockage of the ventricular sodium conductance [13]. In addition, TTX can prolong cardiac conduction time and the left ventricular effective refractory period (ERP). In an isolated heart model of ventricular fibrillation induced by programmed electrical stimulation, TTX can increase the ventricular ERP and conduction time in the infarct zone, in a concentration-dependent manner [12]. Therefore, TTX can be used to control xenobiotic-induced arrhythmias and attenuate ventricular fibrillation. However, it has a narrow therapeutic window and should be used cautiously [14]. For instance, when the dose of TTX was greater than the amount required to block the TTX-sensitive sodium channels in mice, TTX toxicity—which is like respiratory arrest—became prominent, leading to an increased mortality rate [15]. Therefore, the combination of TTX and other agents is drawing continuous attention in many areas, including anti-arrhythmic treatments. Thus, TTX deserves further investigation in the case of arrhythmias, especially in combination with other anti-arrhythmic agents.

Although the individual anti-arrhythmic effects of TTX and LID have been known for decades [16,17], the combination effect of LID and TTX against arrhythmia has not been evaluated. In the current study, a co-formulation of TTX and LID was prepared through freeze drying based on the literature and the results of patch-clamp recording. The effects of the formulation in rats were studied to evaluate the potential of this formulation as an anti-arrhythmic medication.

## 2. Results and Discussion

### 2.1. Inhibition of Nav1.5 Currents by LID, TTX and LID+TTX Mixture

The primary cardiac skeletal muscle sodium channel isoform is Nav1.5 [18]. As shown in Figure 1A,B, TTX and LID have very different structures. Figure 1C shows examples of whole-cell current traces before and after perfusion with LID (100 μM), TTX (100 nM), or an LID (100 μM) + TTX (100 nM) mixture. Currents were evoked by a single 15 ms depolarizing pulse to −10 mV from a holding potential of −130 mV. TTX and LID exhibited different activities on Nav1.5, and the combination of TTX and LID had a stronger inhibitory effect on Nav1.5 than that of either TTX or LID alone (Figure 1D). At molecular level, LID and TTX were reported to interact with voltage-gated sodium channels at different sites [19,20,21]. We inferred that the different structures of TTX and LID led to different activities on Nav1.5, and further led to different antiarrhythmic effects.

### 2.2. HPLC Analysis of LID + TTX Formulations

The content of TTX, LID, and impurities in the LID + TTX formulations were measured through HPLC. Typical retention times for TTX and LID were 22.5–23.5 min and 13–14 min, respectively. The linear interval range of TTX was 0.1024–10.24 μg/mL (r = 1.0000), while that of LID was 0.5010–15.0324 mg/mL (r = 0.9999). The lower limit of quantification for TTX and LID was 0.81 ng/mL and 28.5 μg/mL, respectively.

### 2.3. Preparation and Stability of a Combination Formulation of LID + TTX

TTX is an amino perhydrogenated quinazoline that is slightly soluble in water [22,23], and it is stable in an aqueous solution at pH 2–7. It can be degraded to quinazoline in alkaline solution, leading to a decrease in its activity. Furthermore, TTX degradation increases when the temperature is raised. For instance, 33.63% of TTX was degraded after a 30 d storage at 40 °C. Nonetheless, crystalline TTX powder is more stable than other forms of TTX, with a shelf life of six months at 40 °C, and lyophilized TTX is reported to be shelf-stable and clinically useful [24,25]. As for LID, it is an acetamide that is unstable in the case of acidic or alkaline environments and it can be easily hydrolyzed during production and storage. Therefore, stability should be considered when designing a mixed formulation of LID + TTX.

Some additives can be added to the LID + TTX combination to improve the stability of the formulation. In the present study, several formulations, using mannitol as an excipient, dextran 40 or beta-cyclodextrin as a stabilizer, and citric acid as a co-solvent and a pH modulator, were designed and prepared based on the physical and chemical properties of TTX and LID (Table 1). The stability of these LID+TTX formulations was studied. As shown in Table 2, little difference was found among these formulations at 40 °C, with little change in total impurities. TL001 and TL002 changed in appearance, suggesting that mannitol could maintain the appearance of the formulation. Meanwhile, the total impurities associated with TTX in TL003 increased more slowly than that in TL004, indicating that dextran 40 possessed a better protective effect for TTX when compared with hydroxypropyl-beta-cyclodextrin.

Moisture might have little influence on the LID + TTX formulation, since only a limited change among these formulations at 90% humidity (as shown in Table 2) was observed. TL003, a formulation with excellent stability, was selected as the optimal formulation for further experiments.

### 2.4. The LID + TTX Formulation Significantly Reduced Aconitine-Induced Arrhythmia and Mortality

**Figure 2:**
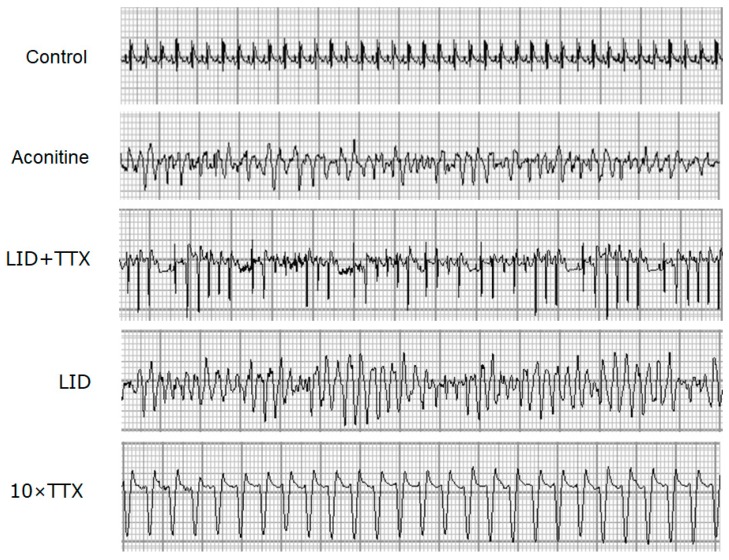
Electrocardiograms (ECG) changes in rats during aconitine-induced arrhythmia. A typical arrhythmia ECG from each group was obtained 10 min after aconitine injection. The lead II ECG was recorded using an RM6240 system, 50 mm/s, 20 mm/mV. The doses used were as follows: Aconitine (55 μg/kg), LID (5 mg/kg), LID (5 mg/kg) + TTX (1 μg/kg), and 10 × TTX (10 μg/kg).

Aconitine induces typical arrhythmia by opening the sodium channel [15]. In accordance with many studies [26,27,28], electrocardiogram (ECG) documented arrhythmias were found in aconitine-injected rats (Figure 2). The protective effects of LID + TTX, LID or 10 × TTX against aconitine-induced arrhythmia were observed in the ECG, when the rats were treated with aconitine for 10 min (shown in Figure 2). Among the treatment groups, the most severe ventricular tachycardia was seen in the rats treated with LID, while only slight arrhythmia was found in the LID + TTX group.

**Figure 3:**
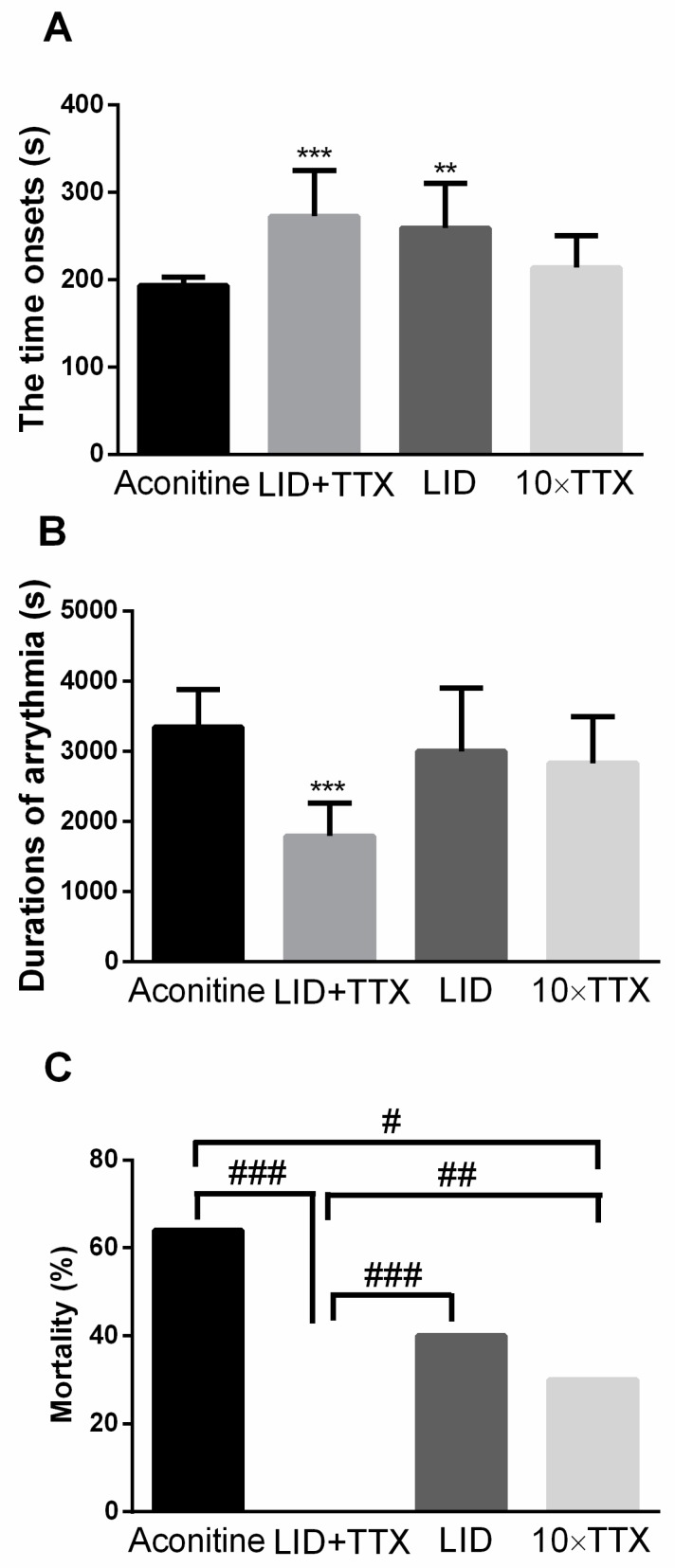
Protective effects of LID + TTX on aconitine-induced arrhythmia. (**A**) LID + TTX effects on aconitine-induced arrhythmia onset and (**B**) duration in live rats. (**C**) LID + TTX improved arrhythmic mortality in Sprague Dawley rats. Data are mean ± SD. Animals were anesthetized using 20% urethane. The doses used were as follows: Aconitine (55 μg/kg), LID (5 mg/kg), LID (5 mg/kg) + TTX (1 μg/kg), and 10 × TTX (10 μg/kg). The data from all tested animals included onset time (n = 20–25), while only data from live animals included duration. Within an observation of 120 min, 16 of 25 rats were dead in the aconitine group, 0 of 24 were dead in the LID + TTX treatment group, 8 of 20 rats were dead in the LID treatment group, and 6 of 20 rats were dead in the 10 × TTX treatment group. ** *p* < 0.01, *** *p* < 0.001 compared with the model group, as assessed using Student’s t-test; # *p* < 0.05, ## *p* < 0.01 ### *p* < 0.001 compared with the model group, as assessed using a χ2 test.

The ventricular arrhythmia is another catastrophic result of aconitine treatment. As shown in Figure 3, with LID + TTX treatment, arrhythmia was delayed and shortened. Evidently, the initial prolongation was a result of LID, because LID can delay the initiation of arrhythmia but 10 × TTX cannot. In addition, the duration of the arrhythmias was determined. The duration of arrhythmias in animals who subsequently experienced sudden death was extremely short. Little significant improvement in the arrhythmic duration was observed in the LID or 10 × TTX group (Figure 3B). It appeared that combining TTX and LID affected the arrhythmic duration, while TTX or LID alone had no such effect. Furthermore, many rats in the model group were killed by aconitine, with a death rate of up to 64%. Amazingly, the administration of LID + TTX decreased the mortality rate of the rats to 0%, while the 10 × TTX treatment reduced the death rate to 30% (Figure 3C). This implies that TTX is more powerful than LID in improving rat survival. Based on these data, we concluded that the combination of LID and TTX was more useful in reducing arrhythmia and mortality in rats when compared with TTX or LID alone.

**Figure 4:**
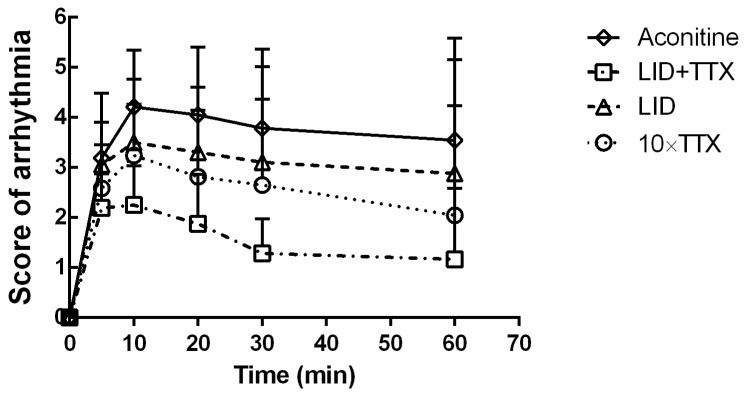
Arrhythmic scores of Sprague Dawley rats during modeling. The doses used were as follows: Aconitine (55 μg/kg), LID (5 mg/kg), LID (5 mg/kg) + TTX (1 μg/kg), and 10 × TTX (10 μg/kg). Data are mean ± SD (n = 20–25).

Arrhythmic scores were measured and analyzed to evaluate the effects of each treatment. As presented in Figure 4, aconitine-induced arrhythmia was severe and rapid. LID + TTX significantly improved this condition (*p* < 0.05, each time point, versus aconitine group) but LID did not (*p* > 0.05, each time point). As for the 10 × TTX group, the arrhythmia scores were obviously reduced after the first 5 min, which also represented the time needed to establish arrhythmias. It was notable that TTX was helpful in slowing arrhythmias, and the combination of LID and TTX might further improve the anti-arrhythmia effects of TTX.

It has been reported that the average dose of LID required to restore normal sinus rhythm is in the range of 2–10 mg/kg intravenously (i.v.) in dogs [29]. Also, 4 mg/kg (i.v.) of LID has been used in rats for analgesic study [30]. In the current study, the LID dose used for rats was set at 5 mg/kg, accordingly. For TTX, 1–20 μg/kg (i.v.) has been used for rats [31], so in our study, the doses used of TTX were set at 1 μg/kg and 10 μg/kg, respectively.

### 2.5. Combination of LID and TTX Significantly Reduced Aconitine-Induced Myocardial Injury

**Figure 5:**
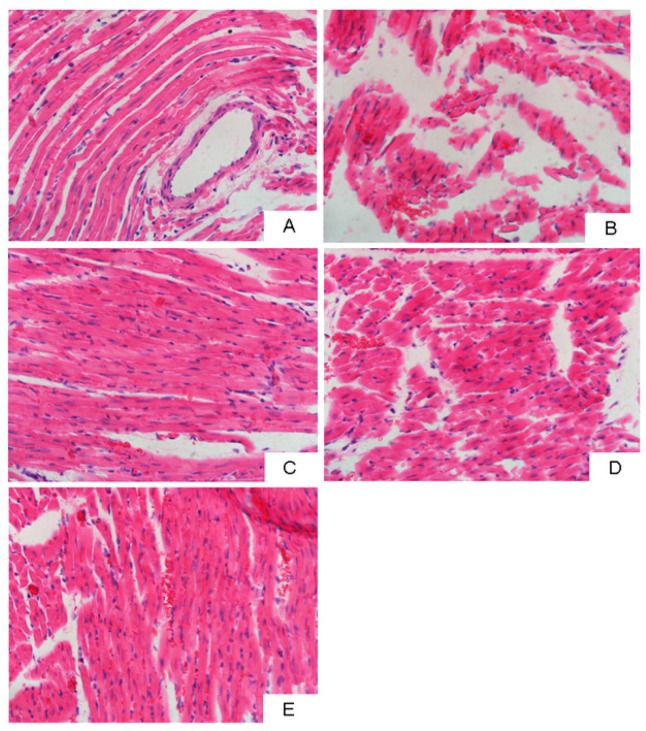
Representative light photomicrographs of left ventricle sections stained using H&E (400× magnification). (**A**) Normal control. (**B**) Model group (aconitine, 55 μg/kg). (**C**) LID (5 mg/kg) + TTX (1 μg/kg) treatment group. (**D**) LID (5 mg/kg) treatment group. (**E**) 10 × TTX (10 μg/kg) treatment group.

To evaluate the influence of LID + TTX on cardiac tissue damage, hematoxylin-eosin (H&E) staining was utilized to determine the myocardial injury caused by aconitine. As shown in Figure 5, only myocyte rupture—rather than inflammation, necrosis, or apoptosis—was observed in each group. Increased areas of myocyte rupture were observed in the aconitine group compared with the control, suggesting that cardiomyocyte rupture caused by aconitine might contribute to death in the rats. However, the administration of LID + TTX, or LID, or 10 × TTX, could retard the myocyte rupture and reduce the bloody areas. Among these groups, the LID + TTX group demonstrated the best outcome in terms of reversing aconitine-generated myocardial injury. This was in accordance with previous results demonstrating that LID + TTX conferred the most powerful cardio protection against aconitine.

Ion channels, including sodium channels, play important roles in arrhythmias. Some channel blockers have been developed as anti-arrhythmic agents. As a Vaughan–Williams Class IB sodium channel blocker, LID is more efficacious than many other anti-arrhythmic agents, but the intrinsic clearance of LID in the liver is very high [32]. Therefore, a single dose of LID for an arrhythmic emergency, administered through intravenous injection, generally reaches 50–100 mg in the clinic. TTX should be classified as a Class IC sodium channel blocker using the Vaughan–Williams classification. TTX produces pure sodium channel inhibition. We expected that a better curative effect could be achieved by combining LID and TTX.

In the current study, LID (5 mg) + TTX (1 μg) exhibited better anti-arrhythmic abilities and less side effects than LID (5 mg) or 10 × TTX (10 μg) alone. Rats treated using LID + TTX obtained better protection against aconitine-induced arrhythmia. More excitingly, LID + TTX eliminated rat deaths caused by aconitine, which has not been previously reported. In addition, using a combination of LID + TTX can reduce the required dosage of TTX. Both male and female rats were included in the current study, but no significant male–female differences were observed. Our data suggest that a combination formulation based on different anti-arrhythmic mechanisms may be a viable approach for fatal arrhythmias.

## 3. Materials and Methods

### 3.1. Drugs and Chemicals

TTX (purity ≥98%) was provided by the Third Institute of Oceanography, Ministry of Natural Resources, Xiamen, China. LID hydrochloride was obtained from Shanxi Xinbaoyuan Pharmaceutical Co., Ltd. (Shanxi, China). Dextran 40 was purchased from Shandong Jinyang Pharmaceutical Co., Ltd. (Shandong, China). Hydroxypropyl-beta-cyclodextrin was purchased from Shandong Xinda Biotechnology Co., Ltd. (Shandong, China). Mannitol was supplied by Qingdao Bright Moon Seaweed Group Co., Ltd., (Shandong, China). Citric acid was obtained from Taishan Xinning Pharmaceutical Co., Ltd., (Guangdong, China). Sodium octane sulfonate was purchased from REGIS Technologies, Inc., (Morton Grove, IL, USA). Aconitine was purchased from West Asia Reagents (Shandong, China). Urethane was purchased from Tianjin Kaixin Chemicals Development Co., Ltd., (Tianjin, China). Acetonitrile (HPLC grade) was purchased from Tedia Company, Inc., (Fairfield, OH, USA.). Phosphoric acid (HPLC grade) was obtained from Chinese Medicine Group Chemical Reagent Co., Ltd. (Shanghai, China). All other solvents were of analytical or chromatographic grade.

### 3.2. Cell Culture

The Chinese hamster ovary cell line stably expressing human cardiac Nav1.5 channels (Qingdao Haiwei Biopharma Co. Ltd., Qingdao, China) was grown in F-12 Nutrient Mixture (Gibco, CA, USA) containing 10% (v/v) fetal bovine serum (Gibco, CA, USA), 100 U/mL penicillin, 100 U/mL streptomycin, and 600 μg/mL G-418 (Gibco, CA, USA) at 37 °C in 5% CO_2_-enriched air. In each experiment, a coverslip was cleaned using the bath solution twice, and then placed on an inverted microscope stage. Nav1.5 currents were recorded using a whole cell patch clamp technique.

### 3.3. Patch Clamp Recording

For patch clamp recordings, the standard bath solution contained 137 mM NaCl, 1 mM MgCl_2_, 4 mM KCl, 10 mM glucose, 10 mM HEPES, and 1.8 mM CaCl_2_. The pH was adjusted to 7.4 using NaOH, and the osmolality was adjusted to 300 mOsm using sucrose. The standard pipette solution contained 65 mM CsCl, 75 mM CsF, 2.5 mM MgCl_2_, 5 mM EGTA, and 10 mM HEPES. The pH was adjusted to 7.4 using CsOH, and the osmolality was adjusted to 300 mOsm using sucrose. LID was dissolved in dimethyl sulfoxide (DMSO) to obtain a stock solution (100 mmol/L). TTX (1 mmol/L, 1% acetic acid solution) was diluted in DMSO to obtain a stock solution of 100 μmol/L. The stock was stored at −20 °C, and then diluted using an extracellular solution to obtain the desired concentration. The concentration of DMSO in the final dilution was less than 0.3% (v/v), and this concentration had no effect on the Nav1.5 currents.

Whole-cell currents were recorded using an Axon 700A amplifier and Clampex software (Molecular Devices, Sunnyvale, CA, USA). Whole-cell patch clamp experiments were conducted at room temperature and with a borosilicate glass microelectrode tip resistance of 1–2 MΩ. After whole-cell recordings were obtained, the liquid junction potentials between the pipette and bath solutions were compensated before the pipette touched the cell. Gentle suction was applied to achieve a gigaseal, and whole-cell access was established. Whole-cell capacitance and resistance were compensated. The current signal was filtered at 8 kHz and sampled at 10 kHz. Cells were stabilized for 5 min after the whole-cell configuration was established and then the currents were recorded. All experiments were carried out at room temperature and the holding potential was −130 mV.

### 3.4. Determination of TTX Content and Impurities

TTX content and impurities were analyzed through HPLC using a method previously reported by Chen et al. with slight modifications [33]. In brief, the analysis was performed using post-column derivation HPLC with a C_8_ column (Agilent ZORBAX SB-C8, 4.6 mm × 250 mm, 5 μm) and a fluorescence detector. The mobile phase was a phosphate buffered solution (PBS) of sodium octane sulfonate (0.3 mL/min) at λ = 365 nm_ex_ and λ = 510 nm_em_. The post-column derivative reagent was sodium hydroxide (4 mol/L) at a flow rate of 0.3 mL/min and the derivative temperature was 110 °C in a 50 μL injection volume.

### 3.5. Determination of LID Content and Impurities

LID content and impurities were determined through HPLC using a method reported by Shan et al. with slight modifications [34]. Analysis was carried out using RP HPLC with a C18 column (TECHMaTe C18-STII, 4.6 mm × 250 mm, 5 μm) and a UV detector. The mobile phase was acetonitrile and PBS (50:50 v/v), and the pH was adjusted to 8.0 using phosphoric acid. Eluents were monitored at 254 nm at a flow rate of 1.0 mL/min and a 5 μL injection volume.

### 3.6. Preparation of the Formulation of TTX and LID

The formulations were prepared using methods previously published by our group with slight modifications [24]. TTX in 10 mL of a 0.1% citric acid solution as solvent was added to LID in 250 mL of water as solvent. The pH of the mixed solutions was adjusted to 3.5–5.0 using 0.1% citric acid. The formulation was ultra-filtered, and the obtained depyrogenated solution was defined as Solution A. Next, mannitol as an excipient and dextran 40 or hydroxypropyl-beta-cyclodextrin as a stabilizer were mixed with water (200 mL) as solvent. Activated carbon was added and stirred at 60–65 °C for 30 min, then filtered. The obtained depyrogenated solution was defined as Solution B. Solutions A and B were mixed and the pH was adjusted to 3.5–5.0 using 0.1% citric acid. Water was added to 500 mL and the solution was filtered through a 0.22 μm microporous filtering film. This filtered solution was added to vials and each vial was vacuum freeze dried to yield the lyophilized powder formulation. The composition of the different formulations is listed in Table 1.

### 3.7. Stability Studies

Sample stability was monitored for 10 d at 40 °C, 75% humidity and 25 °C, 90% humidity. Every 5 d, the content and impurities of the samples were tested.

### 3.8. In Vivo Experiments

Sprague Dawley rats (200–250 g, equal number of males and females) were purchased from Liaoning Changsheng Biotechnology Co., Ltd., (Liaoning, China) and maintained under standard housing conditions. All animal experiments adhered to the principles of care and use of laboratory animals and were approved by the Institutional Animal Care and Use Committee of Shenyang Pharmaceutical University (project identification code: SYPU-IACUC-0427-207).

The rats were anesthetized using 20% urethane (1.2 g/kg, i.p.). Lead II ECGs were recorded using a BD-6240 Biological Data Acquisition & Analysis System (Chengdu Technology & Market Corp., Ltd., Chengdu, China). All tested formulations were dissolved in a sterile saline solution for infusion. The rats were divided into four groups, which were respectively administrated with LID (5 mg/kg, n = 20), LID (5 mg/kg) + TTX (1 μg/kg) (n = 24), 10-fold TTX (10 μg/kg, n = 20), or a formulation without LID and TTX (model group, n = 25, 13 males, 12 females). The tested solutions were administered into the vena jugularis externa (0.1 mL/100 g, i.v.). After stabilization for 2 min, aconitine (10 μg/mL; 55 μg/kg) was administered through continuous infusion into the caudal vein or vena jugularis externa (0.2 mL/min) using an infusion pump (KDS-101, Kd Scientific, Holliston, MA, USA) in all four groups.

Rat ECGs were recorded from arrhythmic initiation, duration, and death (if applicable) and after injection of aconitine, the point at which the first ventricular premature contraction occurred was considered the initial time of arrhythmia. The duration of arrhythmia and death were recorded. Using Curtis–Walker and Mest methods, we assessed arrhythmic severity using six grades to measure outcomes at 5, 10, 20, 30, and 60 min after aconitine treatment. The relative fraction of the most severe arrhythmia was used as an arrhythmia score. Scoring criteria were as follows: 0 points, no arrhythmia; 1 point, atrial arrhythmia, occasional ventricular premature beats; 2 points, early ventricular bigeminy (every other beat), trigeminy (every third beat), frequent premature ventricular arrhythmias, two-degree atrioventricular block; 3 points, short paroxysmal ventricular tachycardia, ventricular premature beats; 4 points, ventricular tachycardia, three-degree atrioventricular block; 5 points, ventricular fibrillation, death.

### 3.9. Histological Assessment

Hearts were fixed in 10% buffered formalin and embedded in paraffin. Histological sections from left ventricle (4-μm thick) were stained using hematoxylin and eosin (H&E). Sections were examined using a light microscope, and photomicrographs were taken (100×).

### 3.10. Statistical Analysis

Data are expressed as mean ± SD and statistical analyses were performed using one-way ANOVA with Dunnett’s post-hoc test. A χ2-test was used to assess arrhythmic deaths. Values of *p* < 0.05 were considered to be statistically significant. For patch clamp recording, the current analysis was performed using Clampfit (Molecular Devices, CA, USA). Data that were fitted to model equations were analyzed using nonlinear regression (Origin, MA, USA).

## 4. Conclusions

In conclusion, a stable LID + TTX combination containing 1 μg of TTX, 5 mg of LID HCl, 6 mg of mannitol, and 4 mg of dextran-40 was developed by freeze drying, which retarded aconitine-induced arrhythmia, attenuated myocardial injury, and reduced the death rate in rats. Its anti-arrhythmic effects were better than those of either TTX or LID alone. This LID + TTX combination may be helpful in treating fatal arrhythmias and reducing mortality.

## Figures and Tables

**Table 1:** Composition of the mixtures used for the formulation of TTX and LID.

Groups	TL001	TL002	TL003	TL004	TL005	TL006	TL007
TTX (mg)	1	1	1	1	0	10	0
LID (g)	5	5	5	5	5	0	0
Mannitol (g)	0	0	6	6	6	6	6
Hydroxypropyl- beta-cyclodextrin (g)	10	0	0	4	0	0	0
Dextran 40 (g)	0	10	4	0	4	4	4

**Table 2:** Stability of formulations at 75% humidity (40 °C) or 90% humidity (25 °C).

Groups		Appearance	Content (%)		Total Impurities (%)	
(40 °C, 75%/25 °C, 90%)			TTX	LID	TTX	LID
TL001	0 day	White, cake shaped	99.16/99.16	99.91/99.91	0.94/0.94	0.10/0.10
	5 days	Partly shrunk/White, cake shaped	98.90/99.13	99.89/99.89	1.15/0.81	0.10/0.10
	10 days	Completely shrunk/White, cake shaped	98.75/99.14	99.90/99.89	1.20/0.81	0.10/0.10
TL002	0 day	White, cake shaped	99.30/99.30	99.89/99.89	0.76/0.76	0.10/0.10
	5 days	Partly shrunk/White, cake shaped	98.61/99.19	99.88/99.89	1.40/0.80	0.10/0.11
	10 days	Completely shrunk/White, cake shaped	98.40/99.24	99.90/99.87	1.67/0.89	0.30/0.11
TL003	0 day	White, cake shaped	98.95/98.95	99.90/99.90	1.32/1.32	0.11/0.11
	5 days	White, cake shaped	98.11/98.76	99.88/99.90	1.74/1.34	0.12/0.11
	10 days	White, cake shaped	97.64/98.64	99.88/99.88	1.87/1.43	0.12/0.12
TL004	0 day	White, cake shaped	99.22/99.22	99.90/99.90	0.96/0.96	0.10/0.10
	5 days	White, cake shaped	98.67/98.66	99.90/99.89	1.39/1.34	0.11/0.11
	10 days	White, cake shaped	97.96/98.56	99.86/99.89	2.35/1.45	0.13/0.11
